# The Heading Weight Function: A Novel LiDAR-Based Local Planner for Nonholonomic Mobile Robots

**DOI:** 10.3390/s19163606

**Published:** 2019-08-19

**Authors:** El Houssein Chouaib Harik, Audun Korsaeth

**Affiliations:** The Norwegian Institute of Bioeconomy Research (NIBIO), Center for Precision Agriculture, Nylinna 226, 2849 Kapp, Norway

**Keywords:** LiDAR-based navigation, local path planner, nonholonomic mobile robots

## Abstract

In this paper, we present a novel method for obstacle avoidance designed for a nonholonomic mobile robot. The method relies on light detection and ranging (LiDAR) readings, which are mapped into a polar coordinate system. Obstacles are taken into consideration when they are within a predefined radius from the robot. A central part of the approach is a new Heading Weight Function (HWF), in which the beams within the aperture angle of the LiDAR are virtually weighted in order to generate the best trajectory candidate for the robot. The HWF is designed to find a solution also in the case of a local-minima situation. The function is coupled with the robot’s controller in order to provide both linear and angular velocities. We tested the method both by simulations in a digital environment with a range of different static obstacles, and in a real, experimental environment including static and dynamic obstacles. The results showed that when utilizing the novel HWF, the robot was able to navigate safely toward the target while avoiding all obstacles included in the tests. Our findings thus show that it is possible for a robot to navigate safely in a populated environment using this method, and that sufficient efficiency in navigation may be obtained without basing the method on a global planner. This is particularly promising for navigation challenges occurring in unknown environments where models of the world cannot be obtained.

## 1. Introduction

A key feature in mobile robots’ navigation is to move safely from a starting point to a defined goal. This safety requires the ability to sense and avoid static and dynamic obstacles in the robot’s path. The interest in path planning for obstacle avoidance in mobile robotics is not new, and the proposed methods can be grouped into two categories: global and local path planning [1]. As for global planning, a general knowledge of the environment is required, for the simple reason that the generated path takes into consideration the model of the world for extracting the optimal path. This path is periodically replanned in order to avoid dynamic obstacles that were not present during the construction of the initial model of the navigating area. Although global methods ensure asymptotically optimal solutions, they are computationally expensive and unsuitable in unknown environments where a model of the world cannot be obtained [2]. In contrast, local path planning relies mainly on sensor readings, which makes the navigation scheme reactive to the observable obstacles by the robot’s sensors. The simplest method in obstacle avoidance using reactive schemes is called the BUG algorithm [3], in which the robot is considered to be a point in space and equipped with tactile sensors. Using the BUG algorithm, the robot follows the contours of an obstacle until a free passage to the target is available. While this method is robust to local-minima problems, it is not optimal when path length is taken into consideration. To overcome this, the authors in [4] presented the TangentBug algorithm that uses range sensors instead of tactile ones. The global heading to the goal is stored, and the robot computes a Local Tanget Graph (LTG) in order to choose an optimal path toward the goal while avoiding obstacles. This method relies at each instance on sensor readings, thus any noise in these readings would directly affect the motion of the robot, resulting in undesirable behavior [5]. To overcome this limitation, another widely used technique for local obstacle avoidance is the Vector Field Histogram (VFH) [6]. It uses the readings from sonar sensors to build a local grid map in Cartesian coordinates. Each cell of the map contains the probability of an obstacle’s presence. Once the local map is generated, a steering vector is then extracted in order to avoid the obstacle while keeping the target as a global heading. The aforementioned techniques can be considered the basis for local obstacle avoidance techniques that came later [5].

The efficiency of obstacle avoidance techniques depends greatly on the fidelity of the sensor readings, as well as the robustness of the path planning method. The most commonly used sensors for detecting obstacles are stereo cameras [7], RGB-D cameras [8], LiDARs [9], and monocular cameras, which are usually combined with machine learning techniques [10]. The LiDARs share with RGB-D cameras the advantage of being robust to lightening conditions, in contrast to stereo or monocular sensors. However, the superiority of LiDARs is mainly due to their wide field of view (FOV) compared with the relatively narrow one of the RGB-D cameras. We considered in this work a 2D LiDAR mounted on the front of a nonholonomic mobile robot.

The advantages of LiDAR sensors made them an appealing choice as a sensory input to gather information about the environment. Their readings are represented as a cloud of points distributed in a planar surface (2D LiDARs), or in the 3D space (3D LiDARs). From this cloud of points, different strategies to detect and avoid obstacles have been introduced. As a first step, most of the works start with defining an obstacle’s location and shape, and then define the best heading to avoid them while navigating to the goal [5]. The simplest method in this direction is based on line extraction [11,12]. Here, the LiDAR’s readings are grouped into small clusters, and each cluster is analyzed in order to define potential segments. The segments are then used for obstacle detection and classification. The authors in [13] extended this idea, sorting the extracted information into two groups: lines and circles. Once obstacles are extracted, the distance between the robot and the center of obstacles may be used for navigation purposes. Similarly, the authors in [14] trained a neural network to estimate the distance to objects based on raw data from a 2D LiDAR. The authors in [15] divided the detected obstacles into three groups. They considered each group to belong to a layer characterized by the distance between the obstacle and the robot, with each of the three layers having its own algorithm for obstacle avoidance and path planning. The authors in [16] took also into account dynamic obstacles. To do so, the readings from a tilted LiDAR were divided into three regions (left, front, and right area). Once an obstacle was detected inside one of the three areas, it was classified as static or dynamic. The dynamic obstacle’s trajectories were then predicted in order to generate a collision-free trajectory for the mobile robot. The authors in [17] used a simpler strategy for obstacle avoidance using a LiDAR sensor. Their method implies that when an obstacle is detected, the avoidance strategy is divided into three steps: turn, pass the obstacle, and turn back to the original heading.

We present in this paper a novel obstacle avoidance method that does not rely on obstacle position or shape estimation. It also deals with the problem of robustness to sensor readings presented in the BUG algorithm, while avoiding the uncertainties in the local map construction presented in the VFH algorithm. The proposed scheme in this paper is straightforward; it takes the immediate readings of the sensors and maps them to velocity space of a nonholonomic mobile robot without any intermediary mapping or obstacle detection and clustering. Furthermore, the new Heading Weight Function (HWF) developed in this paper takes into consideration the summation of the laser beams that include the closest subset of obstacles present within a predefined radius, making it robust to errors in sensor readings, and independent of an obstacle’s shape, location, and nature (static or dynamic). To keep the global heading of the robot toward the target, the HWF is also coupled with the robot’s controller taking into account the target’s position, ensuring that the robot reaches its destination.

The remainder of the paper is organized as follows: we present in Section 2 the proposed approach for robot navigation and obstacle avoidance. Section 3 is dedicated to the obtained results, where we discuss both simulation and experimental results. We conclude the present paper in Section 4 with some future directions and possible enhancements of the proposed scheme.

## 2. Methods

### 2.1. Mobile Robot Kinematics

**Figure 1:**
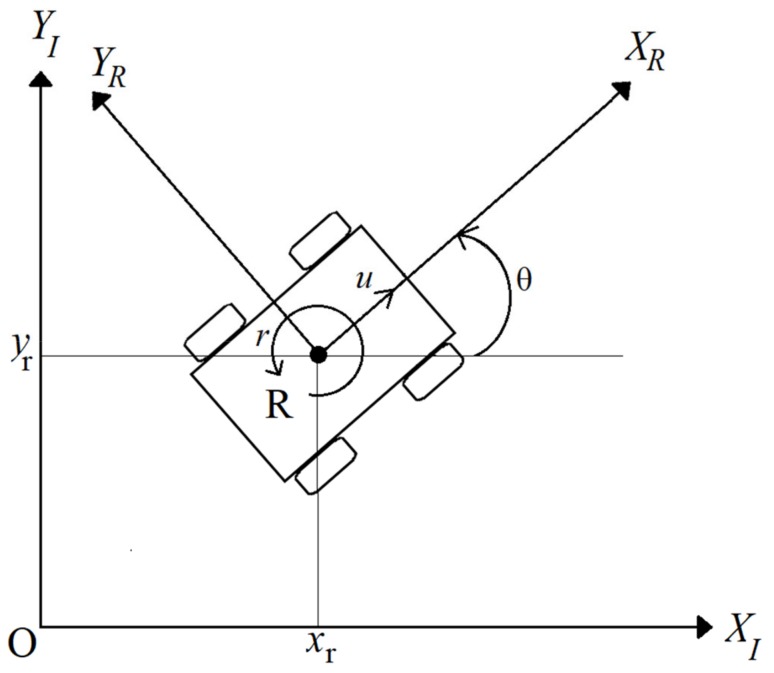
Mobile robot kinematics within a 2D planar surface (see text for symbol and parameter explanation).

The platform included in this work is a nonholonomic, unicycle-like mobile robot (Figure 1). The robot has four wheels, however, on each side, the front and back wheels are linked to each other (front right and back right wheels are linked to each other, and the same for the front left and back left wheels), which makes the robot a differential drive platform. It is considered to perform as a rigid body moving in a 2D planar surface without slipping, and only its kinematic properties are accounted for.

The inertial frame where the mobile robot is located is given by the global reference O: {$X_{I},\;Y_{I}\;$}. *R* is denoted as the mobile robot center and the position of the mobile robot is in the inertial frame {$X_{I},\;Y_{I}\;$}. The heading angle *θ* is the deviation of the mobile robot’s attached frame *{*$X_{R},\;Y_{R}\;$*}* to the inertial frame {$X_{I},\;Y_{I}\;$}.

The position of the mobile robot in the inertial frame can be expressed with the following vector:

(1) $$P_{R}=\;\left[\begin{array}{c} x_{r}\\ y_{r}\\ \theta \end{array}\right],$$

where $x_{r},\;y_{r}\;\;$ are the planar coordinates of the mobile robot in the global reference along {$X_{I},\;Y_{I}\;$}, while θ is the heading of the robot.

The kinematic model of the mobile robot is thus given by:

(2) $$\left[\begin{array}{c} \overset{´}{x}\\ \overset{´}{y}\\ \overset{´}{\theta } \end{array}\right]=\left[\begin{array}{c} \begin{array}{cc} cos\theta & 0\\ sin\theta & 0 \end{array}\\ \begin{array}{cc} 0 & 1 \end{array} \end{array}\right]\left[\begin{array}{c} u\\ r \end{array}\right],$$

where $\overset{´}{x}$ and $\overset{´}{y}$ are the linear velocities in the mobile robot’s frame (**X_R_, Y_R_**), respectively, while $\overset{´}{\theta }$ is its angular velocity. *u* and *r* are to be considered as the control inputs for both linear and angular velocity, respectively.

The robot will be given a specific waypoint **ω**($X_{\omega },\;Y_{\omega }$**)** in its inertial coordinates to navigate to. Since this is a local planner, an assumption is that there exists at least one path from the actual position of the robot P($x_{r},\;y_{r}$) to the goal **ω**($X_{\omega },\;Y_{\omega }$).

The first objective is to ensure that the mobile robot navigates to the desired location in an obstacle-free environment. To this end, let *d* be the Euclidian distance that separates the mobile robot’s position ($p_{R}$) from its destination ($p_{\omega }$). It is expressed as:

(3) $$d=\sqrt{{\left(x_{\omega }-x_{r}\right)}^{2}+{\left(y_{\omega }-y_{r}\right)}^{2}}.$$

The angle *α* is the heading of the robot toward the waypoint **ω**($X_{\omega },\;Y_{\omega }$). It is given by:

(4) α=β− θ,

where β can be written as:

(5) $$\beta =atan2\left(y_{\omega }-y_{r},x_{\omega }-x_{r}\right).$$

To ensure that the robot has arrived at its destination, the following assumption must be fulfilled [18]:

(6) $$\underset{t\to \infty }{\lim}d\left(t\right)=d_{tolerance}\;\underset{t\to \infty }{\lim}\alpha \left(t\right)=0,$$

where $d_{tolerance}$ is a predefined distance that when reached, the robot is considered to have arrived at its waypoint.

Based on our previous work in [19], we saw that the following simple proportional kinematic controller fulfills the requirement of (6), and thus ensures that the mobile robot reaches a given waypoint in an obstacle-free environment:

(7) $$u=\;k_{u}\;d^{2},\;r=k_{r}\;\alpha ,$$

where $k_{u}$ and $k_{r}$ are positive gains.

### 2.2. The Heading Weight Function

**Figure 2:**
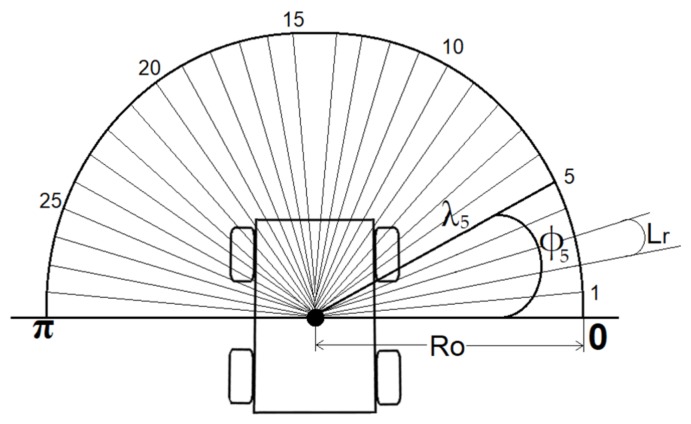
Representation of the Lidar beams on the mobile robot. The actual number of beams is much higher than illustrated here.

The objective of the Heading Weight Function (HWF) is to determine in which direction the robot should navigate when an obstacle is present. The heading should ensure a collision-free path while ensuring that the robot is navigating toward the desired location. Since this is a local planner, the underlying assumption is that there is always a way to reach the goal (until proven wrong), regardless of local-minima problems. A LiDAR was used as an active sensor for obstacle detection. The sensor has a field of view (FOV) of 180 degrees and is located at the center of rotation of the robot R. Figure 2 illustrates the position of the LiDAR sensor on the mobile robot. It should be noted that the figure is just illustrative, and the number of LiDAR beams is shown for explanatory purposes (the actual beam number is much higher, as we will see in the experimental part of this paper).

The LiDAR is a scanning device, which sends beams within its entire FOV range from **0** degrees (defined as the beam being perpendicular to the right of the center line of the robot) to **π** (Figure 2). The angle that separates each beam is called the LiDAR resolution and is denoted in this paper as *Lr*. The LiDAR readings are mapped into a polar coordinate system. Each beam is characterized by variables *Φi* and *λi*, which are the angle and the distance measured of beam number *i*, respectively. *Φi* is calculated as:

(8) Φi = i Lr,

where $i\in \left[0,N\right]$, with *N* being the total number of the LiDAR beams calculated as:

(9) N= π/Lr.

**Figure 3:**
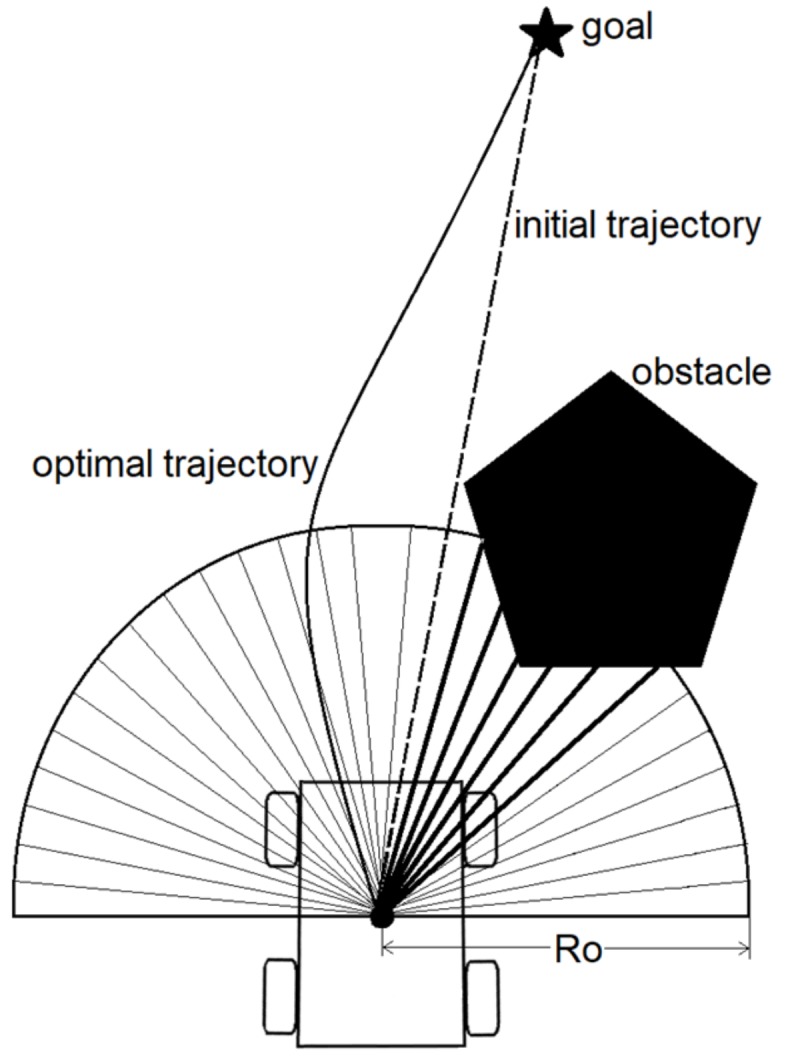
Obstacle presence in the path of the mobile robot.

In the case of the presence of an obstacle in the LiDAR’s FOV (Figure 3), we only take into consideration the LiDAR beams that are lower than a predefined obstacle threshold radius *Ro*. Thus, the HWF includes the LiDAR beams that satisfy the following condition:

(10) $$\lambda i=\left\{\begin{array}{c} \lambda i,\;\lambda i<Ro\\ \infty ,\;\lambda i\geq Ro \end{array}\right..$$

When using the nonlinear controller (7), the initial trajectory that drives the robot from its current position to the goal is a straight line. However, in the case of the presence of an obstacle, as shown in Figure 3, this means that the robot will collide with the obstacle, and to avoid this, the robot must follow an adjusted trajectory that both leads it to the goal and ensures that the robot will not collide with the obstacle.

We propose the initial weight function, where *M* is the number of beams that satisfy (10):

(11) $$Η\left(\Phi ,\lambda \right)=\sum_{i=0}^{M}\sin\left(\Phi i\right)\;sgn\;\left(\cos\left(\Phi i\right)\right)/\lambda i.$$

S*gn(x)* is the signum function, it is denoted as follows:

(12) $$sgn\left(x\right)=\left\{\begin{array}{c} -1,\;x<0\\ 0,\;x=0\\ 1,\;x>0 \end{array}\right.$$

In the initial weight function (11), we take into consideration the summation of multiple terms. First, the sinusoidal result of each beam angle Φi is explained by the fact that the closer the obstacle is to the center of the robot, the higher the sinusoidal result is. This means a higher impact of the initial HWF (11) on the robot’s controllers. Secondly, the result of the sinusoidal term is multiplied by the $sgn\;\left(\cos\left(\Phi i\right)\right)\;\;$ term, simply to know if the beam is on the right or the left side of the robot. Finally, the division on λi, which is the length of the beam number *i*, means that the closer the obstacle is to the robot (the beam length is small), the higher the initial HWF (11) is, which means a higher impact on the robot’s controllers.

The weight value resulting from $Η\left(\Phi ,\lambda \right)$ (11) will thus increase with decreasing distance between the robot and an obstacle (due to a smaller *λ*-value as the denominator) and vice versa. In the case of the example presented in Figure 3, the weight function $Η\left(\Phi ,\lambda \right)$ yields a positive value, since *Φ*_i_ of the most weighted beams are in the range below $\frac{\pi }{2}$, which implies that cos(*Φ*) > 0 and sgn(x) = 1. When added to the nonlinear angular velocity controller, the robot will thus be navigated to the left, making it avoiding the obstacle, and following the optimal path (the safest path while navigating to the target) shown previously. Similarly, if the obstacle is present on the left side of the robot, the result of $Η\left(\Phi ,\lambda \right)$ is a negative value (the most weighted beams are in the range above $\frac{\pi }{2}$), that, when added to the angular velocity controller (7), will lead the robot to the right side.

**Figure 4:**
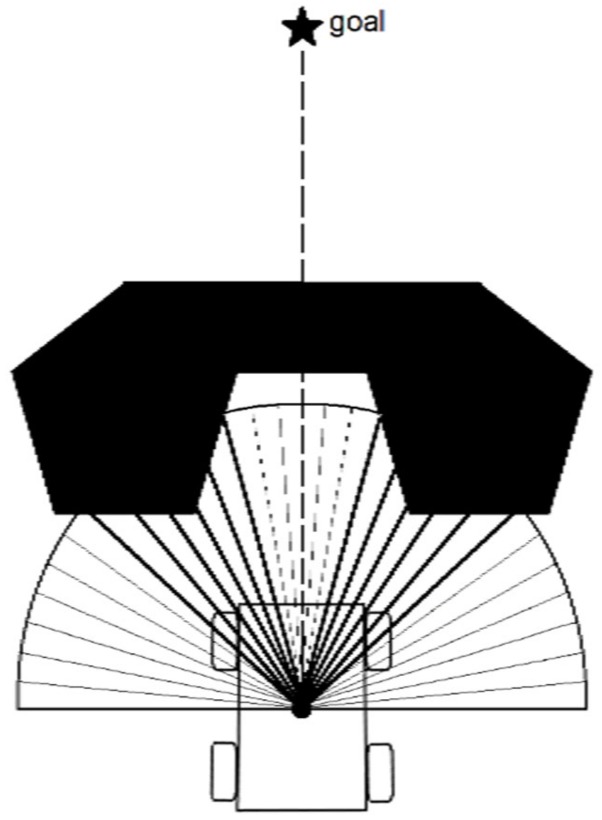
The case of the local-minima configuration.

However, when the robot faces a local-minima configuration, as shown in Figure 4, the result of $Η\left(\Phi ,\lambda \right)\;\;$ is zero, which means that the robot would, if uncorrected, drive straight forward and cause a collision.

To overcome this, we included a free space threshold condition (FST). We evaluated with this condition the free space available in front of the robot to ensure a collision-free passage. The FST constant is related to the physical structure of the robot. It is selected as the necessary space that the robot needs to safely navigate through a tight passage.

**Figure 5:**
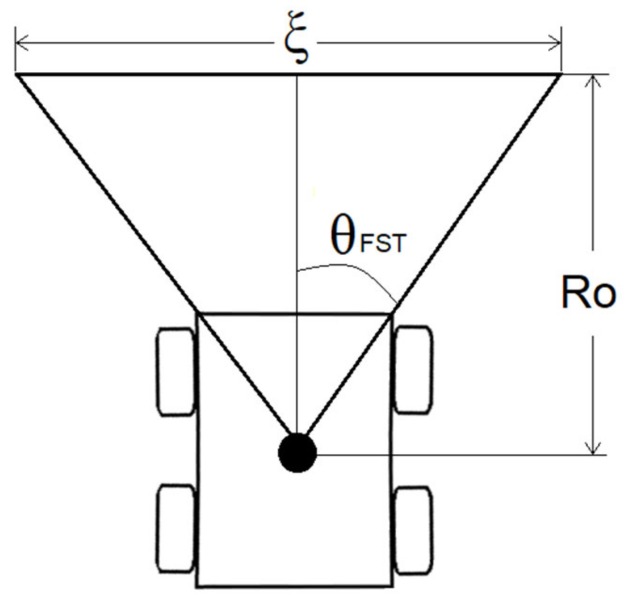
Free space threshold (FST) representation.

Let $\theta_{FST}$ be the predefined angle of the FST condition, and *Ro* is the previously defined obstacle threshold radius. We denote as ξ the value of the LiDAR’s reading of the beams confined in $S\in \left[\left(\frac{\pi }{2}-\;\theta_{FST}\right)/Lr,\left(\frac{\pi }{2}+\;\theta_{FST}\right)\right)/Lr\;$] interval (Figure 5). It is defined as follows:

(13) $$\xi \left(\lambda \right)=\overset{S}{\underset{i=0}{\sum }}\lambda i.$$

The FST condition implies that if **ξ** is lower than FST, then the robot does not have enough space for the passage and needs to turn away in order to avoid the obstacle, while looking for a new passage to the goal. The comparison between FST and **ξ** can be written as:

(14) $$\delta \left(\mathrm{FST},\xi \;\right)=\left\{\begin{array}{c} 1,\;\xi <FST\\ 0,\;\xi \geq FST \end{array}\right.$$

where:

(15) $$\mathrm{FST}=\;2\theta_{\mathrm{FST}\;}\;\mathrm{Ro}\;/\mathrm{Lr}$$

Introducing (14) in (11), the new HWF becomes:

(16) $$\overset{´}{H}\left(\Phi ,\lambda \right)=f^{-1}\left(\delta \left(\mathrm{FST},\xi \;\right)\right)\;Η\left(\Phi ,\lambda \right)+\;\delta \left(\mathrm{FST},\xi \;\right)(k\;\cos\left(\alpha \right)d)$$

**Figure 6:**
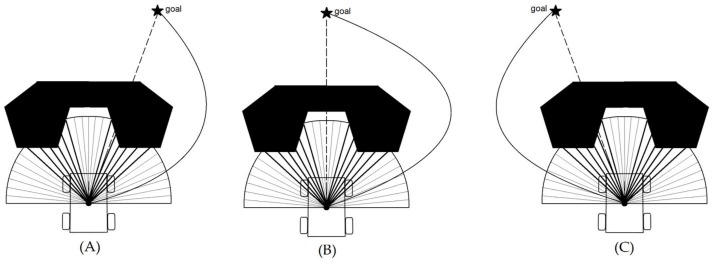
Three cases of nonexistence of FST, with the goal being either slightly to the right of the current course (**A**), straight ahead (**B**), or slightly to the left of the current course (**C**).

In $\overset{´}{H}\left(\Phi ,\lambda \right)$, the first term $f^{-1}\left(\delta \left(\mathrm{FST},\xi \;\right)\right)$ is the inverse function of (14). When the robot has no free passage available in front of it (because of tight space, or the presence of a local-minima situation), it implies that ξ<FST, thus $\delta \left(\mathrm{FST},\xi \;\right)$ = 1, and the term $Η\left(\Phi ,\lambda \right)$ becomes null; this means that the robot needs a way out. Including the inverse function means that $\overset{´}{H}\left(\Phi ,\lambda \right)=\;\delta \left(FST,\xi \;\right)\;(k\;\cos\left(\alpha \right)d)$ where *α* and *d* are the angle and the distance between the mobile robot and its goal, respectively, and *k* is a positive gain. In that case, the HWF drives the robot away with three possibilities, as shown in Figure 6.

In the case that the goal is present to the right of the robot (Figure 6A), the result of $\overset{´}{H}\left(\Phi ,\lambda \right)$ is a negative value, which means that the robot will drive to the right toward the goal. The same happens in the case of Figure 6B. However, in the case of Figure 6C, the result of $\overset{´}{H}\left(\Phi ,\lambda \right)$ is a positive value, which means that the robot will drive to the left toward the goal.

Including (16) in (7), the new nonlinear kinematic control law including the HWF is written as:

$$u=\;k_{u}\;d^{2}\;–\;k_{1}\;abs\left(\overset{´}{H}\left(\Phi ,\lambda \right)\right),$$

(17) $$r=k_{r}\;\alpha +\;k_{2}\;\overset{´}{H}\left(\Phi ,\lambda \right),$$

where $k_{1}$ and $k_{2}$ are positive gains.

The resulted controller (Equation (17)) shows how the HWF is coupled with both linear and angular velocities previously presented in (7). As for the linear velocity, the presence of an obstacle in the path of the robot leads to a decreasing linear velocity, while for the angular velocity, the HWF affects directly the steering behavior of the robot in accordance with the position of the obstacle. We will see in the next section both simulation and experimental results that confirm the efficiency of the presented function.

## 3. Results

### 3.1. Simulation Results

The simulations were carried out using Gazebo [20] and ROS [21]. The environment was a rectangular area, where several obstacles were placed randomly on the surface. The robot had to navigate to the four corners of the area, going from its initial position ($x_{r0},\;y_{r0}$) to w1, then from w1 to w2, from w2 to w3, and finally to w4, where w4 was the same as the initial position of the robot. It should be noted that there was not any a priori information about the environment available for the navigation. Only the simulated LiDAR readings where used in order to create the HWF (16), which was applied to the proposed nonlinear kinematic controller (17). The parameters used during the simulations are given in Table 1.

**Figure 7:**
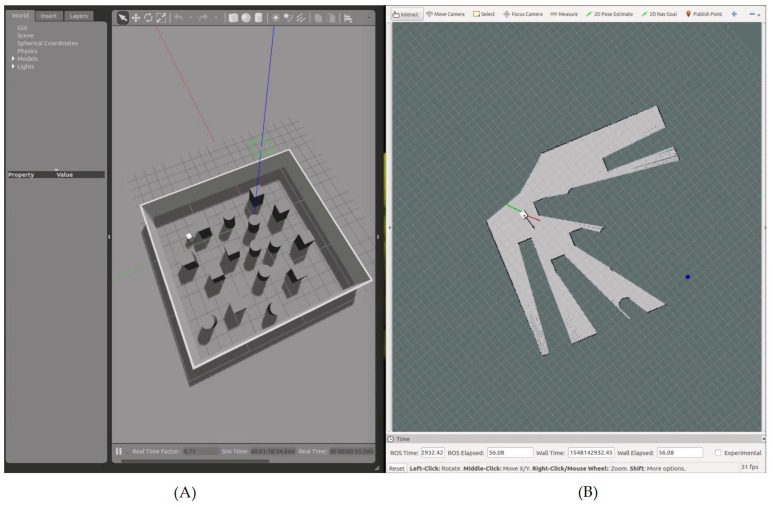
Simulation results showing the robot navigating toward waypoint n1, with the navigation area on Gazebo (**A**), and the simulated state of the robot on Rviz (**B**). On Gazebo (**A**), the red, green, and blue lines represent the 3D axis related to the navigation area. On Rviz (**B**), the robot is shown in white (3D model), and the waypoint is shown as a blue dot. The green line represents the saved path of the robot, the red arrow represents the heading β between the robot and the waypoint in the global frame, and the black arrow represents the actual heading θ of the robot (i.e., the result of using the heading to the waypoint α and the HWF for obstacle avoidance).

Figure 7 illustrates the simulated environment.

**Figure 8:**
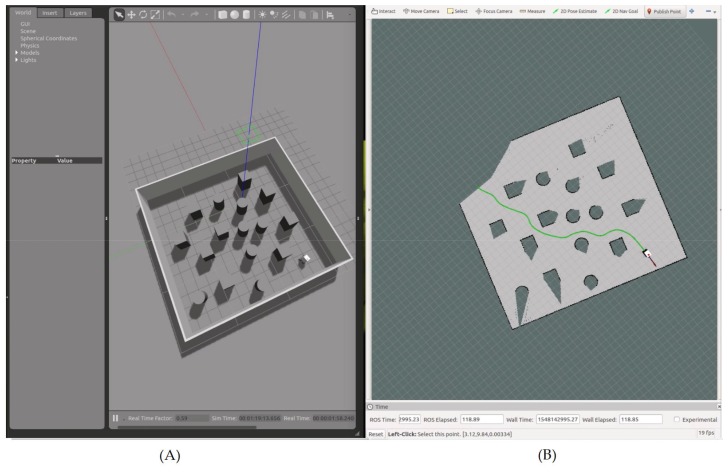
Simulation results showing the path of the robot from its initial location to waypoint n1, with the navigation area on Gazebo (**A**), and the simulated state of the robot on Rviz (**B**).

**Figure 9:**
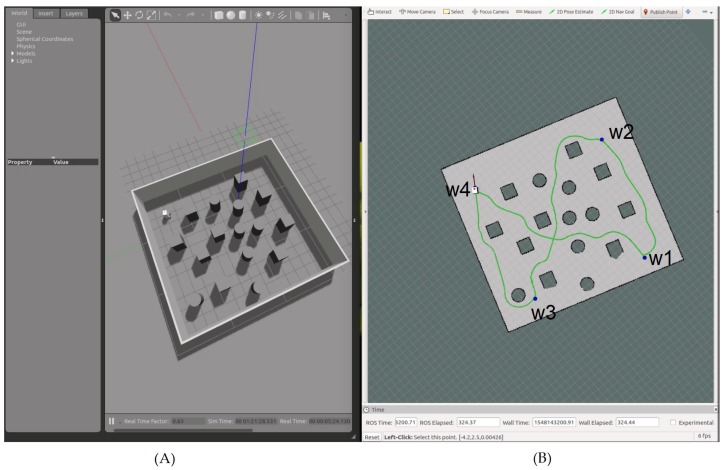
Simulation results showing the global navigation path of the robot, with the navigation area on Gazebo (**A**), and the simulated state of the robot on Rviz (**B**). The waypoints are enumerated according to their chronological order of selection. Every time the robot reaches a given waypoint, a new one is given.

After reaching a given waypoint (Figure 8), another waypoint was selected on one of the four corners of the simulated area (Figure 9).

**Figure 10:**
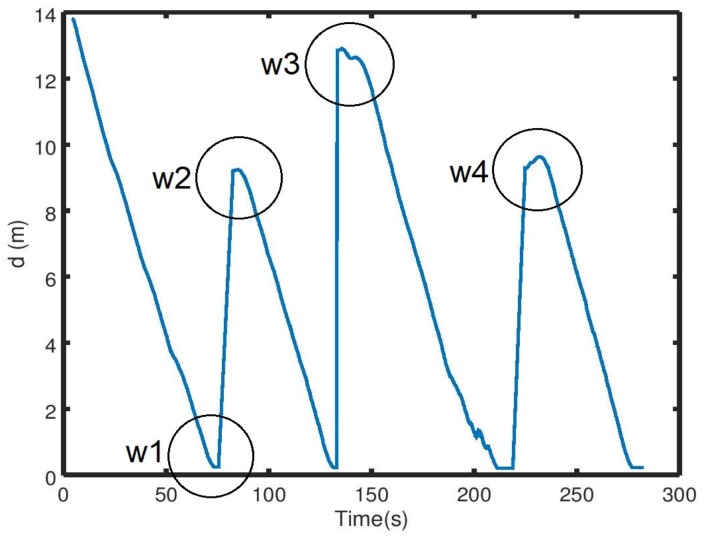
The variation over time of the distance between the robot’s position and the given waypoints.

The variation over time of the distance d is illustrated in Figure 10. We can see that at each time a new waypoint was introduced, a new peak became present, and that the distance decreased over time to the given tolerance distance $d_{\mathrm{tolerance}}$, which means that the first objective of the assumption given in (6) was fulfilled.

**Figure 11:**
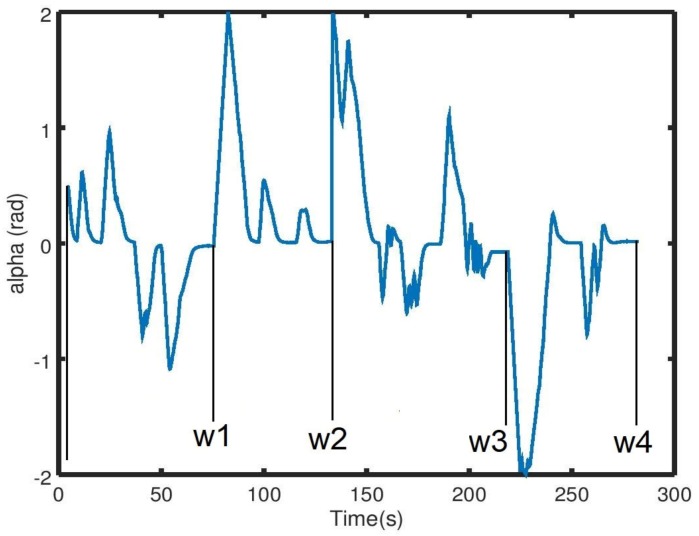
The variation over time of the angle between the robot’s position and the given waypoints.

As for the angle α, the variation over time could be divided into four slots: (($x_{r0},\;y_{r0})$ to w1, w1 to w2, w2 to w3, w3 to w4 (Figure 11), where each slot was the time the robot needed to reach a given waypoint. The angle variations within the slots were a result of obstacles located along the path of the robot between its current location and the waypoint, and the resulting navigation to get around them. Without any obstacles present, this path would have been a straight line. Starting with the transition from the robot’s initial position ($x_{r0},\;y_{r0}$) to w1, the angle α was a positive value, and it decreased over time until the first obstacle was detected, which explains the new peak, meaning that the robot was driving away from the obstacle (Figure 11). However, once the obstacle was out of sight, the robot turned again toward w1, decreasing α to 0, until the presence of a new obstacle (and so on).

We can see from Figure 9 that the robot’s path between the initial position of the robot (which was the same as w4) and w1 was a mixture of right and left turns. This explains the positive and negative peaks present on the first slot of time, which also was the case for the time slots w2–w3 and w3–w4 (Figure 11). This pattern differed, however, for the time slot from w1 to w2. As shown in Figure 9, all the obstacles were present on one side of the path between w1 and w2, which had the consequence that the robot turned only in one direction (to the right), as represented by only positive peaks in the related time slot (Figure 11).

#### The Case of Nonsatisfaction of the FST Condition

**Figure 12:**
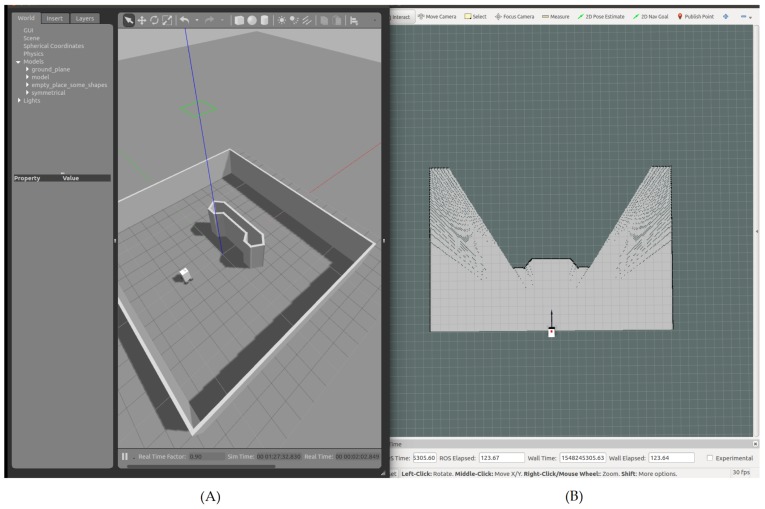
Simulation environment of the nonsatisfaction of the FST condition with the navigation area on Gazebo (**A**), and the simulated state of the robot on Rviz (**B**).

In the presence of a local minima, as illustrated in Figure 4, the FST condition presented in (14) was not satisfied. In order to test the system’s performance under such conditions, we simulated a case with the presence of an obstacle with a local minima between the initial position of the robot and the given waypoint (Figure 12).

**Figure 13:**
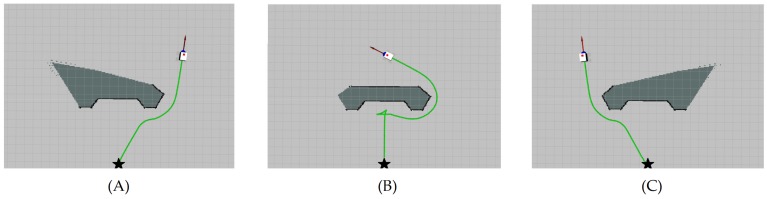
Simulation results of the nonsatisfaction of the FST condition when the selected waypoint is either behind and slightly to the right of the obstacle (**A**), straight ahead and behind the obstacle (local-minima configuration) (**B**), or behind and slightly to the left of the obstacle (**C**).

The simulations started with the robot located at an initial position (−2, 0, 0). Then, we tested the system’s performance for three different locations of a given waypoint. The waypoint was either located behind and slightly to the right of the obstacle, straight forward and behind the obstacle, or behind and slightly to the left of the obstacle (Figure 13A–C).

From Figure 13A, the coordinates of the waypoint were (7, 3.8). We can see that the robot drove in a straight line until the obstacle was within the *Ro*; once detected, the robot deviated from the original path and continued toward the waypoint once a clearance was found on its way. The case shown in Figure 13C is similar to case (A) but with different coordinates for the waypoint, which were (7, −3.8). As for case (B) (Figure 13B), the robot faced the local-minima configuration, where the waypoint (7, 0) was situated on the other side of the obstacle, on the same axis of the initial position of the robot, while the robot’s initial orientation was facing that waypoint. From the path shown in Figure 13B, we can see that once the obstacle was within *Ro*, it found no available space for navigation. Due to the minus sign added to the HWF for the linear velocity input presented in (17), we can see that the robot drove back while changing its direction. The reason for this behavior is that we had a case where there wasn’t any free passage available for the robot, and this implied that ξ<FST, thus $\delta \left(\mathrm{FST},\xi \right)$ = 1. The HWF then became $\overset{´}{H}\left(\Phi ,\lambda \right)=\;\delta \left(\mathrm{FST},\xi \right)\;(k\;\cos\left(\alpha \right)d)$. By subtracting the new HWF from the linear velocity *u* input, as presented in (17), the linear velocity of the robot became a negative entity, which led the robot to drive backward. At the same time, the robot changed its direction because the angular velocity *r* presented in the same Equation (17) was directly influenced by the HWF. The coupled movement (driving backward and turning at the same time) drove the robot out from the local-minima configuration, where the robot was no longer facing the target, and the obstacle was then located to its left. The robot resumed its navigation as shown in Figure 13b to finally reach the given waypoint.

The results of the simulation configurations tested showed that the proposed controller (17) was able to drive the robot from its current location to its destination, thereby satisfying the control objective (6). The video of the simulation results is available online.

### 3.2. Experimental Results

The developed local planner was tested in a real environment by utilizing our customized nonholonomic, unicycle-like robot that drives at a maximum speed of 2.3 m/s. More details about the hardware architecture of the robot can be found in [19].

**Figure 14:**
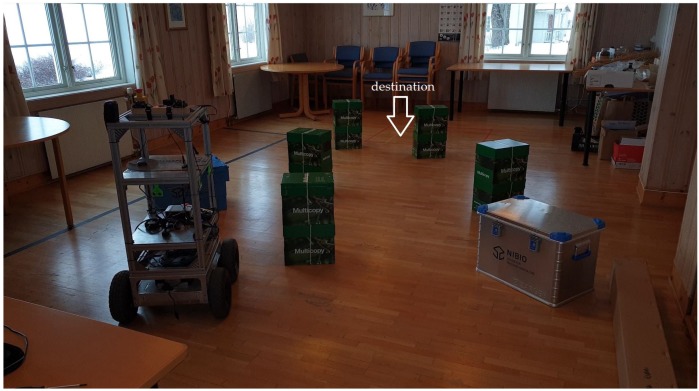
Experimental setup.

As the experimental playground, we chose one of the meeting rooms at our research facility. We randomly placed several boxes in the middle of the room as static obstacles (Figure 14). The robot was then given the task to drive from the starting position to the given destination (waypoint), and thereby navigate past obstacles in the way.

The robot was not equipped with wheel encoders, thus odometry was not an option to estimate the robot’s position inside the room. Instead, we used an ultrasound-beacon-based indoor localization system from [22].

Since the navigation scheme was performed in a two-dimensional space, two static beacons were enough to estimate the 2D position of the robot. The heading of the robot was estimated using the internal compass of the robot.

The parameters used during the experimental results are given in Table 1.

It is worth mentioning that the problem between the speed of the robot and obstacle detection and avoidance can be faced when the frequency of data acquisition, data analysis, and control loop is lower than the speed of the robot. In our case, the loop frequency of the aforementioned processes was 20 Hz, while the maximum speed of the robot in our experiments was limited to 2.3 m/s, thus the predefined obstacle threshold was sufficient to ensure obstacle detection and avoidance at the maximum speed of the robot. The parameters used during the experimentations are given in Table 2.

**Figure 15:**
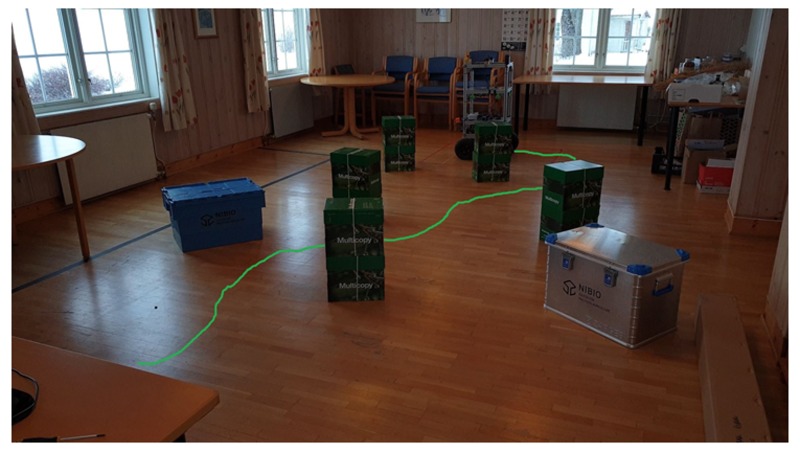
Experimental results showing static obstacle avoidance. The mobile robot traveled across the room past randomly placed boxes to a given waypoint. The virtual overlaid green line on the ground represents the path of the robot.

As a first step, the robot was given the task to navigate in an environment with static obstacles only. Figure 15 shows the robot’s path moving from an initial position to its destination. The green path shown in Figure 15 is a virtual overlaying of the robot’s estimated path on the floor inside the image. We can see that the robot was able to navigate successfully past the obstacles and park at its predefined destination.

**Figure 16:**
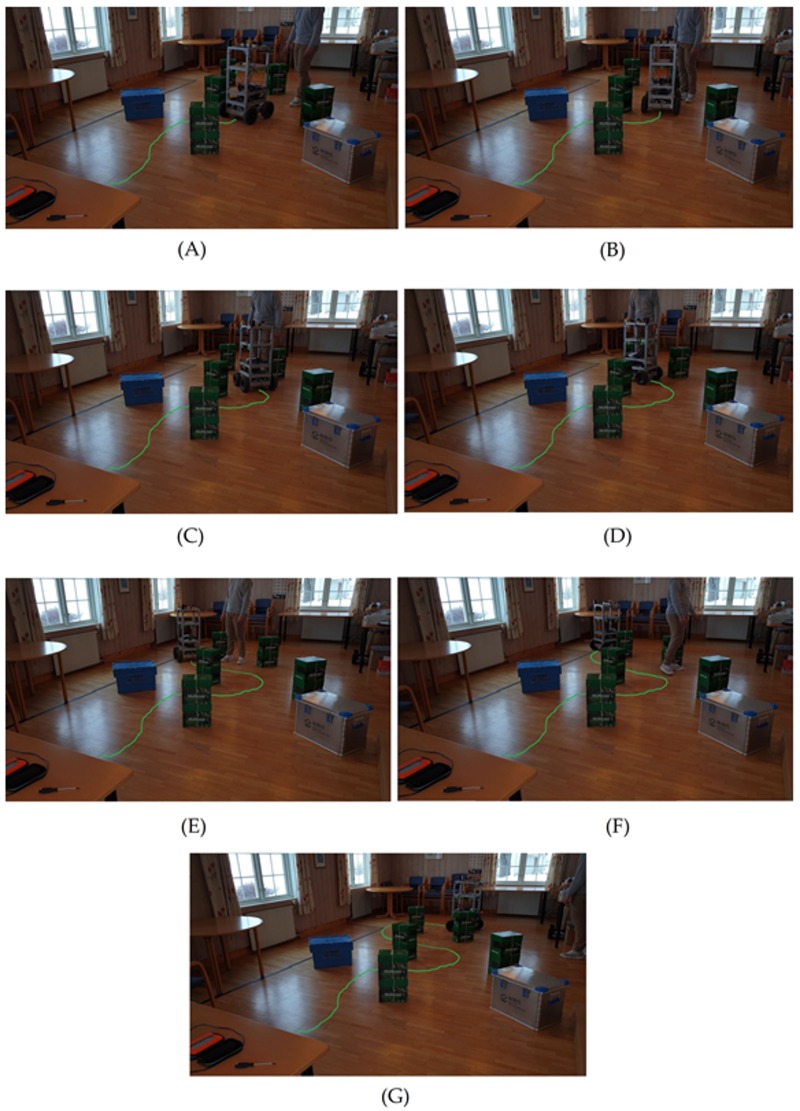
Experimental results showing static and dynamic obstacle avoidance. In (**A**), the human enters the scene and keeps obstructing the robot’s path (**B**–**D**). In (**E**), the human stops obstructing the path and leaves the scene in (**F**). In (**G**), the robot is parked at its final destination. The virtual overlaid green line on the ground represents the path of the robot.

The second step was to test the efficiency of the HWF in the presence of a dynamic obstacle. To do so, we kept the same setup as in the first step (same environment and configuration of the static obstacles). Then, during the robot’s navigation from the starting position to the waypoint, a human entered the scene and obstructed the robot’s path (Figure 16).

The results of the human, dynamic obstruction are shown in detail in Figure 16A–G, in which each subfigure represents a moment of a time sequence: The human entered the scene (Figure 16A) and the robots started deviating (Figure 16B). The human kept on obstructing the robot actively by positioning himself in the current path of the robot (Figure 16C,D). Then, the human stopped the obstruction (Figure 16E), and the robot had to pass an obstacle on its right before turning to its destination (Figure 16F) and parking at the destination (Figure 16G). A video of the experimental results is available online.

The experimental results confirmed the findings from the simulations, that the proposed HWF provides an efficient and robust method to navigate in an indoor environment with static obstacles. Moreover, the tests performed in a real environment showed that the approach could handle situations with human interference as dynamic obstacles.

## 4. Conclusions

We presented in this paper a novel Heading Weight Function (HWF) as a local planner for a nonholonomic mobile robot. A LiDAR was used to detect the presence of obstacles in the robot’s environment. The readings from the LiDAR were given as input to the HWF, which defined the best heading for the robot to avoid obstacles by weighing the LiDAR beam (i.e., of each side of the obstacle). We further included a free space threshold condition (FST) to enable the robot to find a solution in the presence of a symmetrical configuration (left and right weights are equal), or when facing a local-minima configuration. The efficiency of the HWF was demonstrated both by simulations and in real experiments, and the results showed that the robot was able to navigate toward the targets while avoiding all obstacles included in the tests. These findings thus show that even without a global planner, it is possible for a robot to navigate safely in a populated environment. Our method developed is particularly promising for navigation challenges occurring in unknown environments where models of the world cannot be obtained.

The work presented in this paper can be further enhanced by adding a LiDAR on the back of the robot or deploying a 360-degree LiDAR. Since information in three dimensions possesses a larger potential in obstacle detection than information in two, a future research challenge will be to generalize the method in a way to enable the use of 3D information, such as that obtained from stereovision systems, RGBD cameras, or 3D LiDARs.

## Figures and Tables

**Table 1:** Simulation parameters used to test the HWF.

Parameter	Significance	Value
$\;x_{r0}\;$	Initial *x* position of the robot in the global frame	1.92 m
$\;y_{r0}\;$	Initial *y* position of the robot in the global frame	6.93 m
$\;\theta_{0}\;$	Initial orientation of the robot in the global frame	−2.84 rad
$\;d_{tolerance}\;$	Distance tolerance to the waypoint	0.3 m
$\;k_{u}\;$	Positive gain for the linear velocity	0.4
$\;k_{r}\;$	Positive gain for the angular velocity	1.8
$\;k_{1}\;$	Positive gain 1 for the HWF	0.01
$\;k_{2}\;$	Positive gain 2 for the HWF	0.04
Ro	Obstacle threshold radius	1.2 m
Lr	LiDAR resolution	0.004914 rad
$\;\theta_{\mathrm{FST}\;}\;$	Predefined angle of the FST	0.5838 rad
k	Positive gain for the HWF	5.0

**Table 2:** Experimental parameters used to test the HWF.

Parameter	Significance	Value
$\;x_{r0}\;$	Initial *x* position of the robot in the global frame	0.08 m
$\;y_{r0}\;$	Initial *y* position of the robot in the global frame	7.18 m
$\;x_{p}\;$	*x* position of the parking location in the global frame	−0.5 m
$\;y_{p}\;$	*y* position of the parking location in the global frame	1.92 m
$\;\theta_{0}\;$	Initial orientation of the robot in the global frame	−0.08 rad
$\;d_{tolerance}\;$	Distance tolerance to the waypoint	0.15 m
$\;k_{u}\;$	Positive gain for the linear velocity	0.5
$\;k_{r}\;$	Positive gain for the angular velocity	2.2
$\;k_{1}\;$	Positive gain 1 for the HWF	0.001
$\;k_{2}\;$	Positive gain 2 for the HWF	0.04
Ro	Obstacle threshold radius	0.95 m
Lr	LiDAR resolution	0.005817 rad
$\;\theta_{\mathrm{FST}\;}\;$	Predefined angle of the FST	0.5838 rad
k	Positive gain for the HWF	1.2
